# Molecular Characterization of the Llamas (*Lama glama*) Casein Cluster Genes Transcripts (*CSN1S1*, *CSN2*, *CSN1S2*, *CSN3*) and Regulatory Regions

**DOI:** 10.1371/journal.pone.0124963

**Published:** 2015-04-29

**Authors:** Alfredo Pauciullo, Georg Erhardt

**Affiliations:** 1 Department of Agricultural, Forest and Food Sciences, University of Torino, Grugliasco, Italy; 2 Institute for Animal Breeding and Genetics, Justus Liebig University, Gießen, Germany; University of South Florida College of Medicine, UNITED STATES

## Abstract

In the present paper, we report for the first time the characterization of llama (*Lama glama*) caseins at transcriptomic and genetic level. A total of 288 casein clones transcripts were analysed from two lactating llamas. The most represented mRNA populations were those correctly assembled (85.07%) and they encoded for mature proteins of 215, 217, 187 and 162 amino acids respectively for the *CSN1S1*, *CSN2*, *CSN1S2* and *CSN3* genes. The exonic subdivision evidenced a structure made of 21, 9, 17 and 6 exons for the αs1-, β-, αs2- and κ-casein genes respectively. Exon skipping and duplication events were evidenced. Two variants A and B were identified in the αs1-casein gene as result of the alternative out-splicing of the exon 18. An additional exon coding for a novel esapeptide was found to be cryptic in the κ-casein gene, whereas one extra exon was found in the αs2-casein gene by the comparison with the *Camelus dromedaries* sequence. A total of 28 putative phosphorylated motifs highlighted a complex heterogeneity and a potential variable degree of post-translational modifications. Ninety-six polymorphic sites were found through the comparison of the lama casein cDNAs with the homologous camel sequences, whereas the first description and characterization of the 5’- and 3’-regulatory regions allowed to identify the main putative consensus sequences involved in the casein genes expression, thus opening the way to new investigations -so far- never achieved in this species.

## Introduction

The Andean highlands, especially the Altiplano of southeast Peru and western Bolivia, is the natural habitat of South American camelids (llama, guanaco, alpaca and vicuna). These species belong to Camelidae family (together with dromedary, bactrian and wild bactrian camels) and they are members of Lamini tribe, which originated in North America during the Eocene about 40–50 Ma ago [1]. In its autochthonous regions, llamas (*Lama glama*) are kept as a multipurpose animals. Their valuable fleece are obtained by shearing their coarse wool [2], whereas for their ability to carry burdens llamas are used as working animals to haul loads over the mountains. These animals are of major economic and cultural importance for the rural population of Bolivia, Peru, parts of Chile, Argentina and Ecuador. Despite their potential to live on marginal resources in austere environment, llamas have not been exploited as an important food source. For instance, contrary to the Old World camels (dromedary and bactrians), there is no historic tradition of milking llamas and there are many gaps in the scientific literature with regard to the genetic basis of milk protein.

Milk proteins and the corresponding coding genes have been deeply studied in ruminants, whereas such information is still limited in camels and almost completely lacking in llamas. The main component of milk proteins are caseins (αs1, β, αs2 and κ), which are coded by single autosomal genes (*CSN1S1*, *CSN2*, *CSN1S2* and *CSN3*, respectively) clustered in a DNA stretch of about 250 kb, mapped on chromosome 6 in cattle, sheep and goat [3]. In ruminants, caseins have been very well characterized at both DNA and protein level. Goats and cows represent the most polymorphic species, for which many alleles associated with different rates of protein synthesis have been identified [4–6] and at least one allele associated to a “null” content of the corresponding protein have been found [7]. In dromedary camels the β- and κ-casein genes have been fully characterized and polymorphic sites with potential influence on gene expression have been identified [8, 9], whereas a partial genomic DNA sequence for αs1-casein was reported by Shuiep et al. [10].

In alpacas (*Vicugna pacos*), the whole genome has been recently completed (http://www.ensembl.org/Vicugna_pacos/Info/Index), but preliminary sequences are available only for 3 (αs1: ENSVAG00000004344; β: ENSVAG00000004350 and κ: ENSVAG00000003971) out of 4 casein encoding genes. In addition, the low coverage assembly and the tentative annotation built on the human genome led to errors, like for the exon 3 of β-casein which is out-spliced in human [11] and therefore not annotated in the alpaca sequence. This observation supports the need to gain more experimental data to help the annotation of new investigated species like that belonging to Camelidae.

In this respect, unlike what has been accomplished in the aforementioned species, casein genes in llama have not received attention so far. Studies on llama milk were limited to determining fat, protein, lactose or macro-minerals content [12, 13] and recently a proteomic approach allowed the characterization of all casein fractions at amino acid level [14]. With the exception of these examples, no further information is available so far at DNA or cDNA level for casein genes cluster in llamas.

The knowledge of these data is fundamental for future comparative evolutionary studies, to understand the molecular basis of milk protein diversification among the Camelidae and to allow a correct annotation of future genome projects in llamas. Therefore, a deep investigation was undertaken in llamas to explore the genes transcripts of the complete caseins cluster (*CSN1S1*, *CSN2*, *CSN1S2*, *CSN3*) and characterize at cDNA level the corresponding correctly assembled sequences. Comparison with camel genome and cDNA sequences was achieved to establish correct exon boundaries and to investigate the inter-specific genetic diversity, respectively. In addition, the promoter and the 3’ flanking regions of each casein gene were analyzed.

## Material and Methods

### Ethics statements

The study was done according to the German Animal Welfare Law (released on 05/18/2006, last changes on 07/28/2014). On the basis of this law, no notification or approval by the Animal Protection Unit of the Regional Council of Giessen (Germany) was necessary for this study.

The study was carried out on private land and the owner of the land gave permission to conduct it on this site. No specific permissions were required for these activities with the exception of the rules of the afore mentioned German Animal Welfare Law, which were strictly followed. No endangered or protected species were involved in this study.

In the respect of the German Animal Welfare Law, small aliquots of about 5 ml of milk were collected during the routine milking procedure carried out by the owner of the llama farm. The sampling procedure was specifically approved as part of obtaining the field permit.

### Sample collection and Nucleic Acid Isolation

Two unrelated lactating llamas reared in Niedersachsen State (Germany) and belonging to one farm were considered in this study. The animals, approximately in the age of 5 years and at the third lactation, were comparable for type of feed, diet, feeding level and lactation stage (4th month). Milk samples were collected to extract nucleic acids. Total RNA was isolated from milk somatic cells using InviTrap Spin Cell RNA Mini Kit (Stratec Molecular GmbH, Berlin, Germany), whereas the genomic DNA was isolated according to NucleoSpin Tissue (Macherey-Nagel, Düren, Germany).

RNA and DNA concentrations and OD_260/280_ ratios of the samples were measured with the Nanodrop ND-1000 Spectrophotometer (Thermo Fisher Scientific Inc., Waltham, MA, USA).

### Reverse transcription, PCR and cloning

The reverse transcription of total RNA was performed by using an oligo dT_18_. The mix were set up in a final volume of 20μl by using Verso Reverse Transcriptase (Thermo Fisher Scientific Inc., Waltham, MA, USA) according to the standard protocol recommended by the firm.

Primer sequences for *CSN1S1*, *CSN2*, *CSN1S2*, and *CSN3* cDNA amplification (Table 1) were design by means of DNAsis-Max ver. 3.0 software (Hitachi) using as preliminary template the complete sequence of dromedary camel *CSN2* and *CSN3* genes [8, 9] and the bactrian camel genome sequence available in gene bank (EMBL acc. no. AGVR01039100.1). Additional couples of primers were designed on the new sequences determined in the course of the study for llamas *CSN1S1* cDNA (*Exon 11 for*: 5’-AAGTTGTTTCCAGTACCAC-3’ and *Exon 19 rev*: 5’-CACCACTGTGGCATAAC-3’) and *CSN3* cDNA (*Exon 1 for*: 5’-GGCCAACTCAACCTACT-3’ and *Exon 4 rev*: 5’-GTTGGTTGTTCCTGGTTTTG-3’) in order to evidence exon skipping in the transcripts of these two genes.

**Table 1:** Sequences and annealing temperature of the primers used for the characterization of *Lama glama* casein genes cluster.

Gene	Region covered	Position	Primer	Sequence	Ta(°C)
*CSN1S1*	5’-flanking region	Promoter	Forward*	5’-GGGAATCTTATTGATGTAACAGT-3’	60.5
		Exon1	*Reverse*	5’-GGGAAGAAGCAGCAAACT-3’	
	cDNA	Exon 1	Forward*	5’-AGTTTGCTGCTTCTTCCC-3’	61.0
		Exon 21	Reverse*	5’-ATGGCAGTTACAGGAGAAG -3’	
	3’-flanking region	Exon 21	*Forward*	5’-AGTATGAAGGCCACCAAATA-3’	61.5
		3’-end	Reverse*	5’-CTCTGTTCCCACACCTTT-3’	
*CSN2*	5’-flanking region	Promoter	Forward*	5’-GTTTCTCCATTACAGCATC-3’	60.0
		Intron 1	*Reverse* ^†^	5’-TCAAATCTATACAGGCACTT-3’	
	cDNA	Exon 1	*Forward* ^†^	5’-TTCACTTCTTTTCCTCCAC-3’	62.0
		Exon 9	*Reverse* ^†^	5’-TAGTTTATTGAAGTGACTGGT-3’	
	3’-flanking region	Exon 9	*Forward*	5’-GGCACCTTTTCAATCTTT-3’	61.4
		3’-end	Reverse*	5’-TGTAATAGTTTAGCTGAGAT-3’	
*CSN1S2*	5’-flanking region	Promoter	Forward*	5’-AACTAATATGAGAGCTGAG-3’	60.5
		Exon1	*Reverse*	5’-TGAAGGGAAGACAAGTA-3’	
	cDNA	Exon 1	Forward*	5’- CACTGCCTGGACTACTT-3’	60.5
		Exon 17	Reverse*	5’- TCAAAGAGTAGTGAAAACATTTTC-3’	
	3’-flanking region	Exon 17	*Forward*	5’-AGACTGTGCAGAATATTTCC-3’	60.3
		3’-end	Reverse*	5’-ATGTGAACTGGGAGGAG-3’	
*CSN3*	5’-flanking region	Promoter	*Forward* ^*#*^	5’-CACAAAGATGACTCTGCTATCG-3’	62.0
		Intron1	*Reverse* ^*#*^	5’-AGAAGTTAGCCCTCCACAT-3’	
	cDNA	Exon 1	*Forward* ^*#*^	5’-GGCCAGCTCAACCTACT-3’	55.0
		Exon 6	*Reverse* ^*#*^	5’-GCATTCAATTAGCTTTATTA -3’	
	3’-flanking region	Exon 6	*Forward*	5’-GTCTTGCTGAAACCAAA-3’	56.5
		3’-end	Reverse*	5’-ATTCCCGAGAGCAATTT-3’	

Asterisks refer to primers designed on ferus camel genome sequence available in gene bank (EMBL acc. no. AGVR01039100.1); crosses and hashes indicate primers designed on camel *CSN2* (EMBL acc. No. HG969421) and *CSN3* (EMBL acc. No. HE863813) sequences respectively, whereas the other primers were designed on newly determined llamas cDNA sequences.

A typical PCR reaction mix (50 μl) comprised: 50 ng of total cDNA, 1X PCR Buffer (Promega, Madison, WI, USA), 2.5 mM MgCl2, 5 pmol of each primer, dNTPs each at 200 μM, 1 U of *Taq* DNA Polymerase (Promega). PCR was performed under the following thermal conditions: 95°C for 4 min, 35 cycles at 95°C for 45 s, annealing temperature according to the amplicon (Table 1) for 45 s, 72°C for 60 s, and the final extension at 72°C for 5 min. The amplified products were analysed by electrophoresis on 1.5% agarose gel in 0.5X TBE buffer and stained with Midori Green Advance (Nippon Genetics).

The pGEM-T Easy Vector (Promega) was used for cloning individually the cDNAs of each casein transcript. The ligation products were transformed into JM109 High-Efficiency Competent Cells (Promega) following the manufacturers’ guidelines. White recombinant clones were randomly chosen and screened by PCR using standard vector primers M 13. Briefly, each clone was dissolved in 40μl of distilled water, boiled in water bath for 10 min and centrifuged at 14000 rpm for 2 min. 2μl of the supernatant was directly used as template for PCR amplification. The reaction took place in 20 μl of mix using the same chemical and thermal conditions aforementioned, with the exception of the PCR annealing carried out at 56°C for 45 s. A small portion of the product was run on a 1.5% TBE agarose gel to determine the size of the insert. Twenty positive clones from the screening of each casein gene transcript were then randomly chosen for the following sequencing reaction.

### DNA amplification conditions and sequencing

Promoters and 3’ flanking regions of the caseins genes (*CSN1S1*, *CSN2*, *CSN1S2* and *CSN3*) were amplified and sequenced in the investigated llamas. A set of 8 primers were designed (Table 1). Standard PCR reactions were accomplished in a final volume of 50 μl containing 100 ng of genomic DNA in a mix having the same chemical condition already reported before. The thermal profile of the PCR reactions was set up as follows: 95°C (4 min), 35 cycles at 95°C (60 s), annealing temperatures depending on amplicon (Table 1) (45 s), 72°C (90 s), final extension at 72°C (10 min).

All PCR products (both from cloning screening and from regulatory regions) were purified using MSB Spin PCRapace kit (Invitek, Germany) and sequenced in both directions using BigDye chemistry (Applied Biosystems) by ABI 3130 Genetic Analyzer (Applied Biosystem).

### Bioinformatics

Homology searches, comparison among sequences, and multiple alignments were accomplished using DNAsis-Max ver. 3.0 software (Hitachi Software). The genome sequence of *Camelus bactrianus* (EMBL acc. no. AGVR01039100.1) was used to establish exons subdivision, whereas the sequences of *Camelus dromedarius* [15] were used to describe differences in the structure of the casein transcripts and to detect inter-specific genetic diversity. Preliminary sequences of alpaca genome (*Vicugna pacos*) were excluded from the analysis because at the present the contigs are still fragmented and casein genes are limited to 3 (αs1, β and κ) out of 4 sequences. The putative transcription factor binding sites were searched by Transfact 7.0 software considering 85% as minimum binding score. Prediction signals and combined cleavage sites for leader peptides was determined by SignalP V4.1 software (www.cbs.dtu.dk/services/SignalP), whereas NetPhos server 2.0 (http://www.cbs.dtu.dk/services/NetPhos/) was used for the prediction of phosphorylation sites considering 0.9 as minimum rate score for post-translational modification. In addition, manual identification of phosphorylated Ser was accomplished according to the recent discoveries on Fam20C casein kinase which recognizes the S-x-E motifs [16].

## Results and Discussion

### Analysis of the casein cluster transcripts and genes structure

The transcripts of the caseins genes cluster (*CSN1S1*, *CSN2*, *CSN1S2* and *CSN3*) were isolated from two lactating llamas. A total of 288 positive clones (72 clones for each gene for the two investigated llamas) were analysed by PCR and randomly chosen for sequencing after the evaluation of their length by gel electrophoresis.

**Fig 1:**
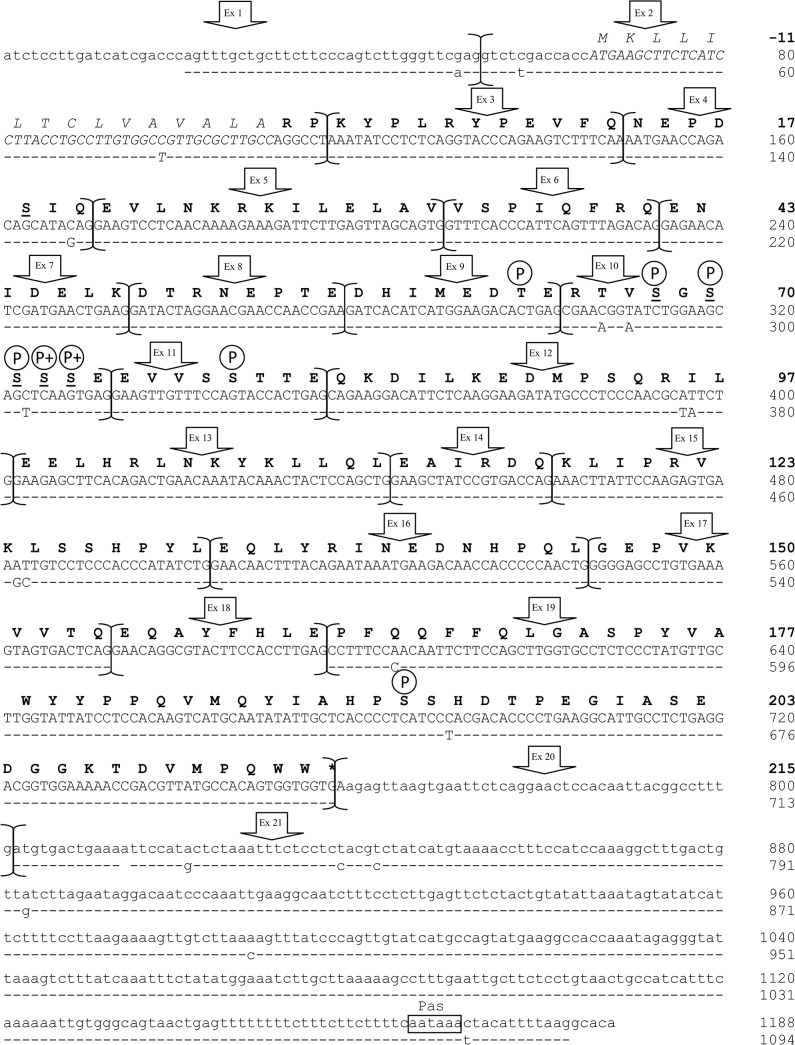
Lama glama complete CSN1S1 cDNA (αs1-casein). Complete cDNA sequence (EMBL acc. no.: LK999986) and exon subdivision (brackets) of *L*. *glama CSN1S1* (upper line) and comparative alignment with the homologous αs1-casein cDNA of *C*. *dromedarius* (EMBL acc. no.: AJ012628). Dashes represent identical nucleotides to those in upper lines. In lower cases the 5’- and 3’- Un-Translated Regions (UTR), the polyadenylation signal (Pas) is indicated by a box. The corresponding mature protein is reported in bold, whereas the signal peptide is in italics and asterisk represents the termination stop codon. Putative phosphorylation sites predicted with a score over 90% are indicated with P. Phosphorylated serines reported by Kappeler et al. [15] are underlined, whereas the plusses indicate Ser preferentially phosphorylated by Fam20C.

**Fig 2:**
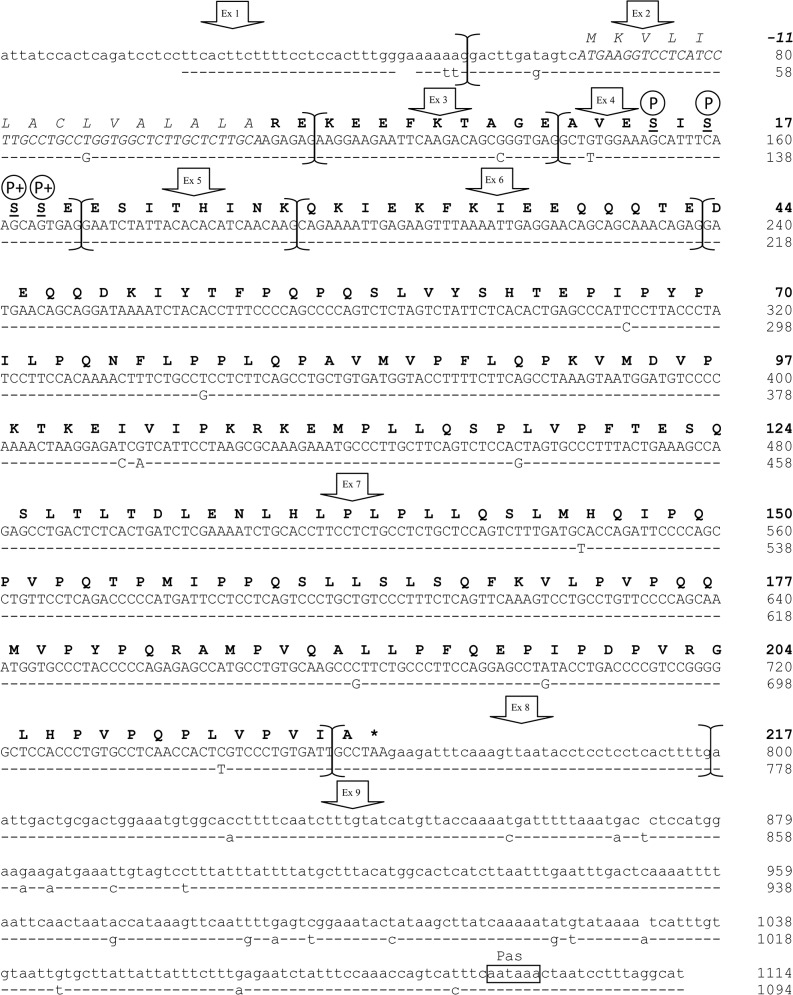
Lama glama complete *CSN2* cDNA (β-casein). Complete cDNA sequence (EMBL acc. no.: LK999992) and exon subdivision (brackets) of *L*. *glama CSN2* (upper line) and comparative alignment with the homologous β-casein cDNA of *C*. *dromedarius* (EMBL acc. no: AJ012630). Dashes represent identical nucleotides to those in upper lines. In lower cases the 5’- and 3’- Un-Translated Regions (UTR), the polyadenylation signal (Pas) is indicated by a box. The corresponding mature protein is reported in bold, whereas the signal peptide is in italics and asterisk represents the termination stop codon. Putative phosphorylation sites are indicated with P. Phosphorylated serines reported by Kappeler et al. [15] are underlined, whereas the plusses indicate Ser preferentially phosphorylated by Fam20C.

**Fig 3:**
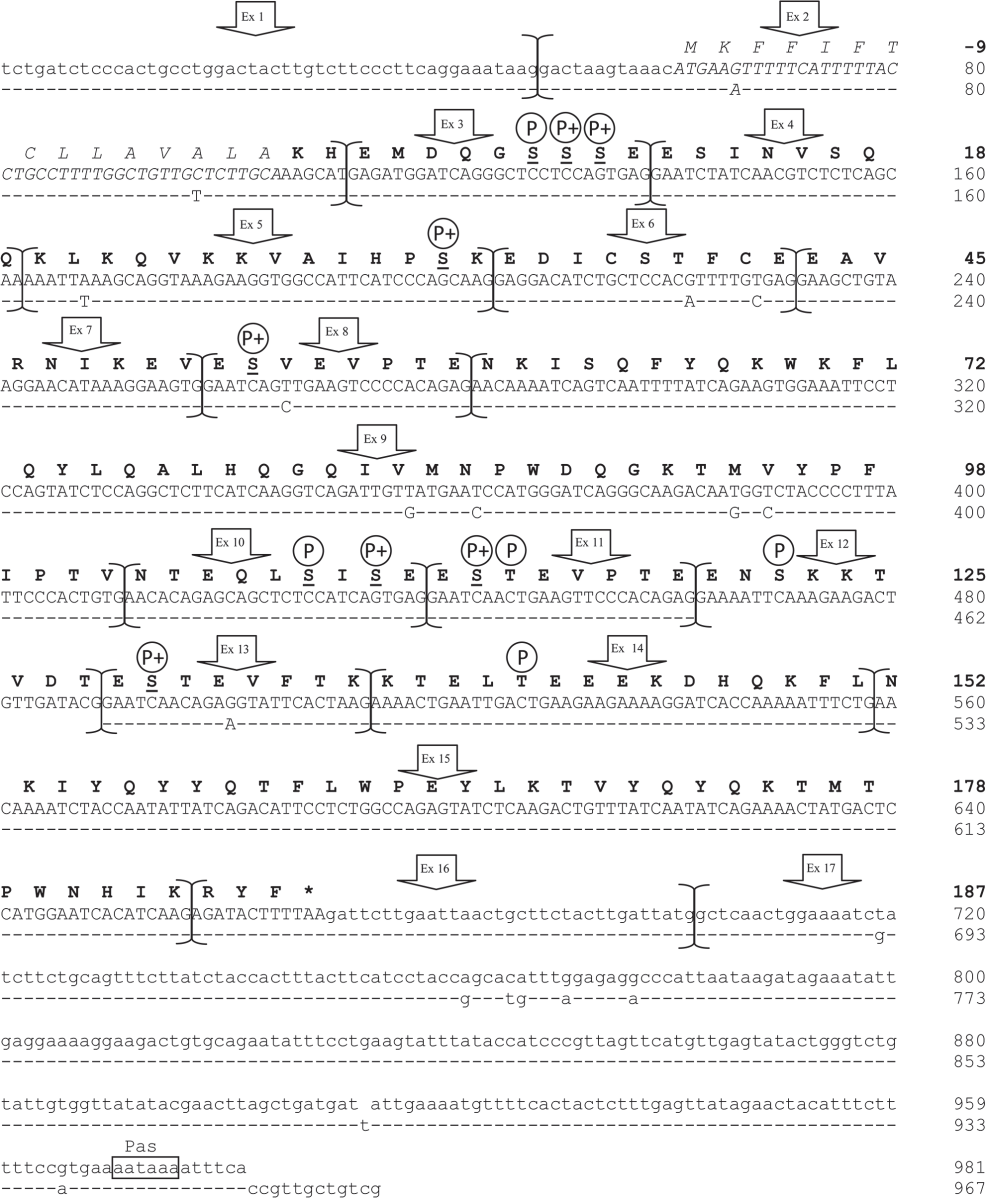
Lama glama complete *CSN1S2* cDNA (αs2-casein). Complete cDNA sequence (EMBL acc. no.: LK999989) and exon subdivision (brackets) of *L*. *glama CSN1S2* (upper line) and comparative alignment with the homologous αs2-casein cDNA of *C*. *dromedarius* (EMBL acc. no.: AJ012629). Dashes represent identical nucleotides to those in upper lines. In lower cases the 5’- and 3’- Un-Translated Regions (UTR), the polyadenylation signal (Pas) is indicated by a box. The corresponding mature protein is reported in bold, whereas the signal peptide is in italics and asterisk represents the termination stop codon. Putative phosphorylation sites are indicated with P. Phosphorylated serines reported by Kappeler et al. [15] are underlined, whereas the plusses indicate Ser preferentially phosphorylated by Fam20C.

**Fig 4:**
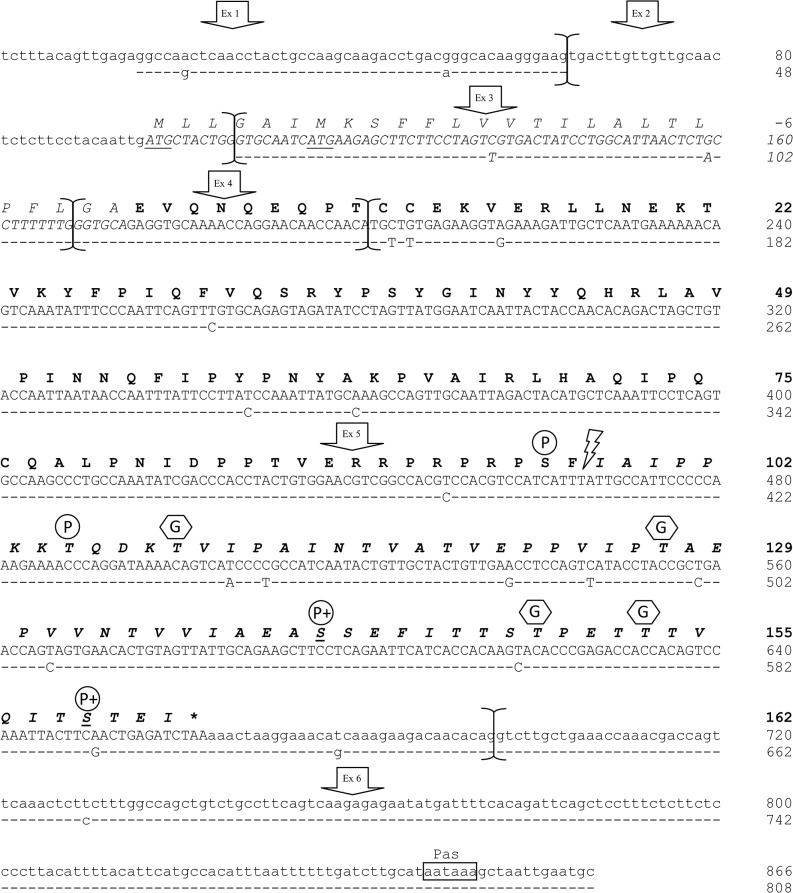
Lama glama complete *CSN3* cDNA (κ-casein). Complete cDNA sequence (EMBL acc. no.: LK999995) and exon subdivision (brackets) of *L*. *glama CSN3* (upper line) and comparative alignment with the homologous κ-casein cDNA of *C*. *dromedarius* (EMBL acc. no.: Y10082). Dashes represent identical nucleotides to those in upper lines. In lower cases the 5’- and 3’- Un-Translated Regions (UTR), the polyadenylation signal (Pas) is indicated by a box. The corresponding mature protein is reported in bold. The chymosin cleavage site (Phe^97^-Ile^98^) is indicated by a flash-arrow, whereas the glycomacropeptide (GMP) 65 amino acids long is reported in bold italics. The signal peptide is in italics and asterisk represents the termination stop codon. P, phosphorylated serine, whereas the plusses indicate Ser preferentially phosphorylated by Fam20C; G, glycosylated threonines.

The most represented populations for each of the gene transcripts were those correctly assembled (in total 245 out of 288 clones, 85.07%). They encoded for mature proteins of 215, 217, 187 and 162 amino acids respectively for the *CSN1S1*, *CSN2*, *CSN1S2* and *CSN3* genes. The homologous genes in dromedary camel encode for casein of the same length with the only exception of the *CSN1S2*, which gives an αs2-casein of 178 amino acids (9 residues shorter than llamas) [15], whereas compared to cattle, the llamas casein show a higher number of amino acids for the αs1-CN (215 *vs* 199 aa) and β-CN (217 *vs* 209 aa), and lower length for the αs2-CN (187 *vs* 207 aa) and κ-CN (162 *vs* 169 aa). The sequences described in Fig 1 and Figs 2–4 were submitted to EMBL with the following IDs: LK999986, LK999992, LK999989, LK999995, and they represent the most frequent genetic variants of llamas milk caseins.

The comparison of the transcripts with the genome sequence of bactrian camel (EMBL acc. no. AGVR01039100.1) allowed the subdivision in exons: 21 for the *CSN1S1* (Fig 1), 9 for the *CSN2* (Fig 2), 17 for the *CSN1S2* gene (Fig 3) and 6 for the *CSN3* (Fig 4). This subdivisions evidenced the extremely split architecture of the casein genes in llamas, which slightly differs from the structure of the homologous genes in ruminants. In fact, taking the bovine casein cluster as reference [6], the corresponding llama genes showed 2 extra exons for the *CSN1S1* (αs1-casein) gene (21 *vs* 19), one less exon for the αs-2 (17 *vs* 18) and one extra exon for the *CSN3* (κ-casein) gene (6 *vs* 5).

This shows also in llama that the organization and orientation of the casein genes is highly conserved, although in different species the molecular diversity of these genes is achieved through variable use of exons [3]. In addition, studies in this field were often produced only using genomic DNA as template for sequencing and not mRNA for transcript analysis, therefore the alignments with already existing reference sequences might be responsible of redundant information, exon losses and errors in gene annotations. This is well exemplified by the β-casein encoding gene in the alpaca genome [14]. Multiple alignment of the amino acid sequence of β-casein from eleven eutherians strongly suggests a species specific out-splicing of exon 3 in the human β-casein gene [11]. As a consequence, since human genome is used as a reference, a putative sequence encoded by exon 3 of the β-casein encoding gene from alpaca is missing as well. However, the DNA sequence encoding for the exon 3 can be easily retrieved approximately 130 bp upstream of the provided *Vicugna pacos* genomic sequence.

### The *CSN1S1* gene transcript (αs1-casein)

Fifty-three out of 72 positive clones (73.6%) analysed for the αs-1 casein in llama showed a gene transcript correctly assembled (1188 bp). It is subdivided in 21 exons ranging in size from 18 bp (exon 14) to 387 bp (exon 21). The first exon (53 bp) plus 12 bp of the exon 2 are not coding at all. The signal peptide (15 amino acids) is coded by the following 45 nucleotides of the exon 2, whereas the mature peptide (215 amino acids) is encoded by the last 2 triplets of the exon 2 and the remaining part of the cDNA. The translation stop codon TAA is realized between the last two nucleotides of the exon 19 and the first bp of the exon 20, whereas the polyadenylation site is located between the 365th and 370th nucleotide of the exon 21 (Fig 1).

The comparison of the whole cDNA with the homologous cDNA in dromedary camel showed that the llama *CSN1S1* contains one extra exon which was not described for the dromedary mRNA [15]. In particular, the exon 20 is 44 bp long in llama and it is partially coding for the termination stop codon because of its first adenine (Fig 1). The analysis of the gene sequence showed an identity of 95% with the exon 18 of the porcine cDNA (NM_001004029) and *CSN1S1* gene (EMBL acc. no. EU025875.1), and a similarity of 91% with bovine (X59856), goat (AJ504710) and sheep (JN560175) homologous gene corresponding to the same exon.

It is well known that the variation in mRNA and protein is primarily due to alternative splicing, duplication, and insertion/deletion events in addition to nucleotide mutations. The transcripts analysis of llama αs1-casein gene showed that 19 out of 72 positive clones (26.4%) were represented by a population of mRNA deleted of the exon 18 as consequence of alternative splicing. This exon is 24 bp long and encodes for 8 amino acids (EQAYFHLE) missing in the shorter protein variant.

**Fig 5:**
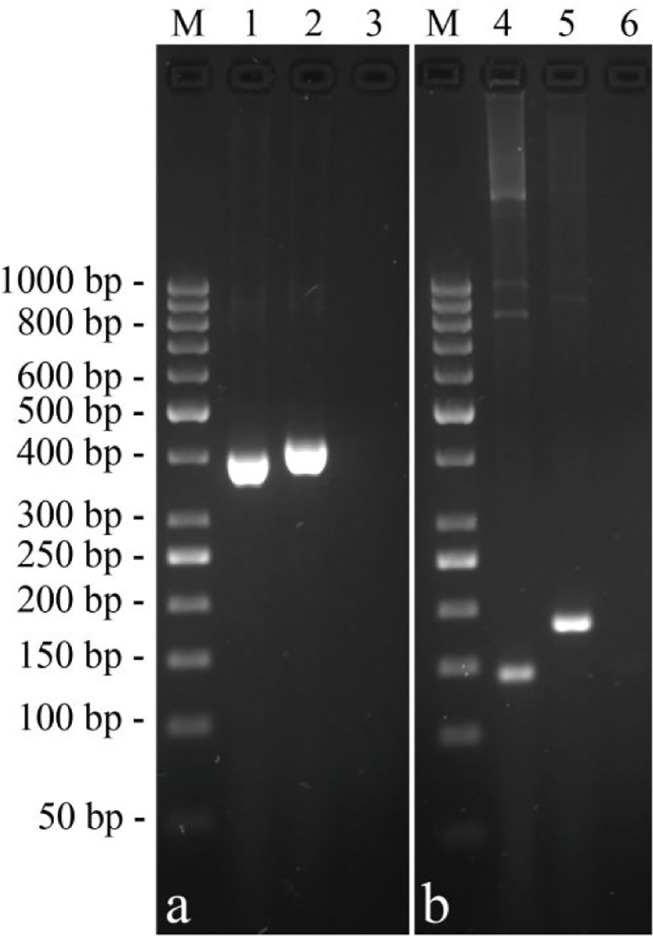
Exon skipping events showed by gel electrophoresis analysis. Gel electrophoresis analysis of the clones showing exon skipping events. Lines 1–2 shorter (397 bp) and longer (421 bp) mRNA variant showing the splice out the exon 18 for the *CSN1S1* (**a**). Lines 4–5 shorter (142 bp) and longer (185 bp) mRNA variant showing the skipping of the cryptic exon 2 for the *CSN3* (**b**).

In order to pinpoint the parts of transcripts with variable length, exon11F/exon19R specific primers were used for cDNA amplification. Two PCR products of different size were obtained (Fig 5A). In the shorter fragment a deletion of 24 bp corresponding to the entire exon 18 was evidenced. These findings agree with the data reported by Saadaoui et al. [14] at protein level, where a difference of about 1,037 Da was evidenced in the molecular mass of two αs-1 casein variants. A similar event was already described by Kappeler et al. [15] for the camel αs1-casein A and B variants, whereas exon skipping events are also known for the exon 16 of camel αs-1 [10]. Long and short variants of αs1-CN also occur in ovine milk as a result of differential splicing of the heterogeneous nuclear RNA [17], as well as it was also showed in goat [7, 18] and cattle where, for instance, the skipping of the exon 4 results in the A variant [19]. Likewise, porcine αs1-casein also showed such a heterogeneity, since multiple forms of porcine αs1-casein cDNA have been described [20]. Therefore, our data confirm also in llama the presence of two variants A (215 amino acids) and B (207 amino acids) as result of the alternative out-splicing of the exon 18 (Fig 5A).

Another genetic event rather frequent among the mammals is the casual use of cryptic splice sites when a glutaminyl residue (Q) is encoded as first amino acid of the exon [21, 22]. A similar site in llama is the first codon of the exon 12 (Q83) and the analysis of the nucleotide sequence at the intron11/exon12 junction confirmed the presence of a cryptic site of splicing (tttttttag/CAG). The occurrence of a competitive AG, downstream from the proximal one, can be used alternatively [23]. Despite that, this event was not detected in the screening of the llama αs1-CN gene transcripts. This might be due to the low number of analysed clones, therefore we can not exclude that this event is common also in llama, but further studies are necessary to better investigate the presence of this codon skipping event also in llama αs-1 casein.

Finally, a possible duplication event occurred between the exon 12 (45 bp) and the exon 15 (42 bp) of llama *CSN1S1* gene. With the exception of the first triplet, these two exons shares more than 65% of their nucleotide sequence, but they encode for different amino acids. It is known that duplication events of many small exons have contributed to the establish the actual structure of *CSN1S1* gene in mammals [24]. However, only the duplication of exon 10 and 13 in cattle [25] and goat [26] are readily detectable at the DNA level (homology 96.2%). On the contrary, the duplication event of the exon 12/exon 14 in human seems to be present in all species, but only in human the level of sequence conservation reach value of 66% at DNA level, whereas the average level of amino acid conservation is lower than 35% [3].

No additional duplication events were detected, although the llama exon 14 (18 bp) shares similar features with the homologous exon in rat and mouse characterised by multiplication events up to 9 and 14 copies respectively [3].

### The *CSN2* gene transcript (β-casein)

All the analysed positive clones for the β-casein showed a gene transcript correctly assembled of 1114 bp and arranged in 9 exons. The exon 5 is the smaller in size (24 bp), whereas the exon 7 is the longer (519 bp). As in the αs1-casein, the first exon (52 bp) of the llama β-casein plus 12 bp of the exon 2 are not coding at all, whereas the signal peptide (15 amino acids) is coded by the following 45 nucleotides of the exon 2. The mature peptide (217 amino acids) is encoded by the last 2 triplets of the exon 2 and the remaining part of the cDNA. The translation stop codon TAA is realized between the 4th and the 6th nucleotide of the exon 8. The polyadenylation site is located at the exon 9 between the nucleotides 294th and 299th (Fig 2).

In llama no splicing events affecting the β-CN were found. This result on gene transcripts only partially agrees with the findings of Saadaoui et al. [14] where milk samples analyzed by LC-ESI-MS showed a protein isoform with a molecular mass corresponding very likely to a β-casein cryptic splice variant resulting from a deletion of a nucleotide triplet encoding a glutamine residue and bearing four phosphate groups.

Furthermore, the llama *CSN2* is not characterised by duplications events and no major rearrangements seem to have occurred at cDNA level, thus confirming the higher degree of conservation between the orthologs, as already reported for other species [3].

### The *CSN1S2* gene transcript (αs2-casein)

The screening of 72 positive clones of the llama αs2-casein revealed only mRNA populations correctly assembled. The sequencing of the randomly chosen clones showed a cDNA made of 981 bp (Fig 3). The comparison with the bactrian camel genome allowed to divide it in 17 exons ranging in size from 24 bp (exon 4, 8, 11 and 13) to 279 bp (the exon 17). Analogously to the αs1 and β-casein, the first exon (48 bp) of the llama αs2-casein plus 12 bp of the exon 2 are not coding at all. Fifteen amino acids encoded by 45 bp of the exon 2 (nucleotides 13–57) represent the signal peptide, whereas the rest of the cDNA encodes for the mature protein of 187 amino acids. The translation stop codon (TAA) is realised between the nucleotides 10–12 of the exon 16, whereas the polyadenylation site is located at the exon 17 (nucleotides 268–273).

One extra exon was found in llama αs2-casein cDNA when compared with the homologous cDNA of dromedary camel (Fig 3). In fact, Kappeler et al. [15] did not describe transcript sequences homologous to llama exon 12, which is 27 bp long and encodes for a peptide of 9 amino acids (ENSKKTVDT). The analysis of the exon sequence showed an homology of 96.3% with the predicted αs2 cDNA of *Pantholops hodgsonii* (XM_005985429), a Tibetan wild antelope well adapted to high altitudes (~3300–5200 m above sea level) whose harsh living conditions and difficult grazing environment remind similar situation of Andean mountains. Slightly lower identity (90%) was shared with the exon 13 of the bovine (M94327.1) and goat (AJ297316.1) *CSN1S2* gene, as well as about 89% with buffalo (FM865619), sheep (GU169085) and horse (NM_001170767) homologous cDNA and 85% with the exon 14 of donkey (FN298386.2) *CSN1S2* I gene.

The gene structure of *CSN1S2* is known to be result of exon replication events in primate, artiodactyla and rodentia [3, 27]. Recently, this was proven also in equids, where the analysis of the donkey *CSN1S2* I cDNA showed a perfect duplication of the exon 10 [28].

The llama αs2 cDNA confirmed this situation by the duplication of several exons. For instance, the exons 15 is the duplication of the exon 9 (68.7% of nucleotide similarity), whereas the exon 8 is replicated in the exons 11 and 13, which showed 87.5% (8 *vs* 11), 70.8% (11 *vs* 13) and 62.5% (8 *vs* 13) of homology at the DNA level and average amino acid similarity of 62.5%. The second amino acid encoded by each of these exons is a phosphorylated serine, as reported also in dromedary camel by Kappeler et al. [15]. A similar event is realised also between the exon 3 and 10 (69.2% of similarity at the cDNA level and 44.4% at protein level) where in total 5 serine are phosphorylated.

The differential use and presence of P-sites was indicated as one of the manifestations of diversification among the species [3]. In this respect, the llama seems to belong to a group of species, including bovine, goat, sheep, camel and pig, which are characterized by only one αs2-like gene with 2–3 major P-sites to ensure the total calcium binding capacity [3].

No splicing events affecting the αs2-casein cDNA were found in our llama samples. This result agrees with the findings of Kappeler et al. [15] for dromedary camels and it confirms the llama milk protein data reported by Saadaoui et at. [14]. However, allelic and non-allelic exons skipping are considered as frequent events in case of αs2-casein gene in different species [28–32].

The extremely split architecture of these genes, the incidence of mutations and the lack of accuracy in the intricate processing mechanism of RNA maturation reflect the occurrence of these splicing events. Considering the nature of the llama *CSN1S2* gene, rearrangements resulting from alternative splicing were expected, therefore the lack of variation in New and Old world camelids αs2 mRNA populations needs further investigation to elucidate the reason for the higher level of conservation.

### The *CSN3* gene transcript (κ-casein)

Length analysis of the 72 positive clones for the llama κ-casein cDNA and the following sequencing results showed the presence of two cDNA populations, 866 bp and 823 bp long in the ratio 66.6% (48 clones) *vs* 33.3% (24 clones) respectively.

These two mRNA populations differ for the presence of an additional exon of 43 bp in the longer variant. Taking as reference the dromedary sequence (Y10082), such insertion event is realised between the exons 1 and 2 of the corresponding camel *CSN3* cDNA (Fig 4). In order to better identify the parts of transcripts with variable length, specific primers (exon1F/exon4R) were used for clones amplification. Two PCR products of 185 bp and 142 bp were obtained for the longer and shorter variant respectively (Fig 5B), thus confirming the presence of the additional transcript sequence.

With the purpose to verify whether the sequence of the additional transcript has similarities with exons of other genes, we compared it with the bovine reference RNA sequences. The comparison showed an average identity of 98% with several genes transcripts, including claudin-12 (*CLDN12*), calcium/calmodulin protein kinase (*CAMK2N1*), and nebulin-related anchoring protein variants (*NRAP*). All these genes encode for proteins that at different levels might share behaviours comparable to that of κ-casein. In particular, they are unable to bind calcium themselves and they use target molecules to facilitate the paracellular conductance and the Ca^2+^ transport in the tissues [33–35]. In addition, the sequences comparison evidenced 46% of similarity with the exon 61 of the *FBN2* gene. This *locus* encodes for the fibrillin 2, a glycoprotein containing several calcium binding epidermal growth factor domains (cbEGF) and belonging to the same gene family of fibrinogen [36] from which the κ-casein gene (*CSN3*) is supposed to be originated by a duplication gene event [37].

The sequence of the extra exon was also compared with the genome sequences available in EMBL to clarify whether it is present also in other species. Similarities were found only with the κ-casein gene of three species: 100% with dromedary camel (HE863813), 94% with donkey (FR822990) and 91% with human (HSU51899), identifying in all cases an intronic region.

**Fig 6:**
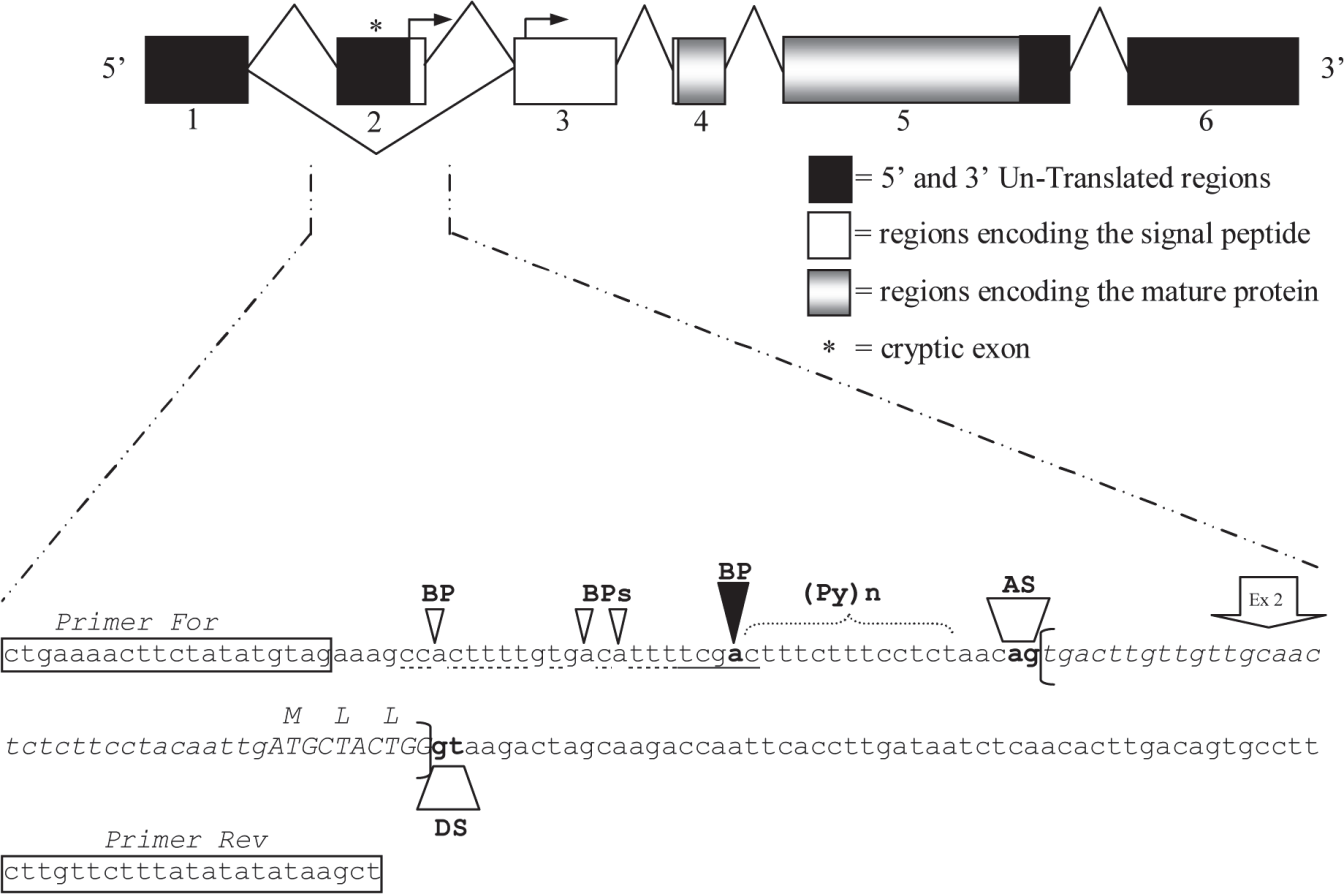
Splicing combinations and cryptic spliceosome mechanism at the κ-casein gene (CSN3) Schematic representation of the aligned exon structure of the llama κ-casein gene (*CSN3*). The top of the figure shows the two possible splicing combinations with (upper) and without (lower) the cryptic exon. The down part of the figure shows in details the sequence of cryptic exon 2 (in brackets) with the respective 5’- and 3’- intronic flanking regions. The branch point site is fully underlined and the bold adenine represent the main branch point (black triangle), followed by a polypyrimidine tract (Py_n_) and the acceptor splice site (AS). The dotted-line and the white triangles represent additional polypyrimidine tracts and alternative branch points, respectively. The donor splice site (DS) for the following intron splicing is normally present.

Removal of intron sequences from pre-mRNA is carried out by the spliceosome machinery, which recognizes specific sites (donor splice site, branch point and acceptor splice site) in a complex molecular mechanism. Any deviation from consensus can result in an overall decreased of affinity for the spliceosome [38]. In order to verify the presence of these sites for the investigated region, specific primers were designed on the homologous dromedary *CSN3* gene (HE863813) and used for the amplification and sequencing of llamas DNA samples (Fig 6). No polymorphic sites were found. According to Zhang et al. [39] the obtained sequence (183 bp) underwent computational splice site prediction (http://www.fruitfly.org/seq_tools/splice.html) which confirmed the presence of the three conserved splicing sequence elements: a branch point, followed by a polypyrimidine tract and a terminal AG acceptor site (score 0.87) at the extreme 3’ end of the intron. Furthermore, the presence of a donor site at the 5’ end of the intron was predicted with a score of 0.99 confirming the occurrence of the additional exon.

This result is very surprising if we consider that the structure of the *CSN3* gene (subdivided in 5 exons) is very well conserved among the species [3]. However, sequence information from a number of independent cDNA clones excluded the possibility of cloning artefacts, whereas the presence of two mRNA variants suggested the “cryptic” nature of this additional exon (Fig 6).

The presence of cryptic exons for the casein genes was already observed in other species. For instance, in human the exon 3 of the β-casein was reported as cryptic [11, 40]. Subsequently, in vitro transcription experiments showed that four purines interrupting the polypyrimidic tract of the intron 2 were responsible for the differential processing of the human β-casein [41]. In llamas, the presence of a extended polypyrimidine tract and the occurrence of alternative branchpoints (BPs) besides the normal mammalian consensus (5’-YTRAY-3’) might be likely considered the cause of the alternative exon skipping.

The presence of a cryptic exon in κ-CN cDNA is also particulary interesting because it adds new reading frame at the 5’-end and it leads to have two possible ATG (Methionine) as translation start codon, one on the cryptic exon 2 and one canonical on the exon 3 (Fig 4). As a consequence, the longer variant of llama κ-CN is characterized by a signal peptide 6 amino acids longer (*MLLGAI*) at NH_2_-terminus, three coming from the cryptic exon 2 and three coming from the reading frame of the following exon. The canonical reading frame is restored at the nucleotide 115 with the second ATG (Methiotine). This condition would allow to have a signal peptide for both derived protein variants and it would guarantee the essential role of κ-CN in the casein micelle stabilization.

The inclusion of the novel esapeptide had no influence on the identification of the signal cleavage site recognized between the amino acids 26–27. The mature κ-casein is encoded by the last 9 triplets of the exon 4 and almost all the exon 5 for a total of 162 amino acids. The stop codon is realised between the nucleotides 460–462 of the exon 5, whereas exons 1 and 6 are not coding at all. The polyadenylation site is located between the 153th and 158th nucleotide of the exon 6 (Fig 4).

### Prediction of post translational modification

The analysis of the Ser/Thr phosphorylation sites predicted by NetPhos server v2.0 and manually, for the identification of Fam20C protein kinase motifs, indicated a total of 28 putative sequence sites with a prediction score over 90% for the software assisted method. In particular, they occur eight times in the αs1-CN (7 Ser at 68, 70, 71, 72, 73, 79 and 192 and one Thr at 63), four times in the β-CN (Ser at 15, 17, 18 and 19), twelve times in the αs2-CN (Ser at 8, 9, 10, 32, 53, 108, 110, 113, 122, 130 and two Thr at 114 and 141) and four times in the κ-CN (3 Ser at 96, 141, 159 and one Thr at 105) (Figs 1–4).

In general this prediction confirms the data found by Kappeler et al. [15] on dromedary camel and Saadaoui et al. [14] on llama, however some exceptions occurred. For instance in the αs1 casein, Ser 18 was indicated as phosphorylated in dromedary camels [15], while in llama the same amino acid residue had a prediction score of only 0.836. On the contrary, Ser at 79 and 192 and Thr at 63 showed higher rate of 0.994, 0.977 and 0.983, respectively (Fig 1). Manual identification of Ser-Xaa-Glu consensus motif indicated that Ser72 and 73 might be preferentially phosphorylated by Fam20C Golgi-enriched fraction casein kinase (GEF-CK). This protein kinase belongs to atypical family of kinases localised within the Golgi apparatus and it is involved in the phosphorylation of several secreted proteins implicated in biomineralization, including caseins and the small integrin-binding ligand, N-linked glycoproteins (SIBLINGs) [16]. In the αs2 casein, the third triplet of the llama exon 12 (not described in camels) encodes for the Ser 122, which is putatively phosphorylated with a score of 0.922. Seven Ser out of 10 followed the Fam20C motifs confirming the phoshorylation sites indicated by Kappeler et al. [15] in dromedary camels. Furthermore, two Thr at 129 and 156 showed a rate of 0.949 and 0.932 respectively. Although the phosphorylation of threonine in the Thr-Xaa-Asp motif is relatively uncommon for caseins [42], we can not exclude that such a phosphorylation occurs. For instance, Thr 66 was partially phosphorylated in cattle αs2-casein C [43], whereas recently Li et al. [44] identified Thr 56 as a new phosphorylation site of cattle β-casein. In particular, the latter amino acid (corresponding to llama Thr 42) falls within a stretch of 9 residues well conserved in camels [9] and herein also found as putatively phosphorylated with a score of 0.844.

A threonine putatively phosphorylated (score 0.929) is present also in the κ-CN at position 105, whereas a second phosphorylation motif (score 0.970) was revealed at Ser 96 (Fig 4). No further phosphorylation motifs were found by NetPhos server. This is in contrast with the findings of Kappeler et al. [15], who identified two phosphorylated Ser at 141 and 159 (Fig 4). However, the different position of phosphorylation sites in llamas appears to be controversially interesting. In fact, on the one hand, Ser 96 is next to the cleavage site of the chymosin (Phe 97—Ile 98) and a post-translational modification in this position might have a negative steric hindrance on the enzyme action (Fig 4). On the other hand, the same amino acid phosphorylated belongs to the insoluble para-κ-casein, therefore after the cleavage it might promote positively the coagulation and the stabilization of the curd by the electrostatic interaction with the Ca^2+^ during the digestion.

Conversely, in dromedary camel both phosphorylated Ser (141 and 159) belong to the glycomacropeptide (GMP), which is soluble in water. These two Ser are conserved also in llamas, therefore phosphorylation events might likely occur also in these positions. In fact, although not indicated by NetPhos, these two Ser fall within a Fam20C GEF-CK Ser-Xaa-Glu motif, thus confirming the putative phosphorylation of these sites and the importance of a dual approach (bioinformatic and manual) for this analysis.

In general, these data confirm the complex heterogeneity and the potential variable degree of phosphorylation also in llama casein. Considering the contrasting information on camelids casein [14, 15] and the data of the present work, further investigations are necessary for elucidating the different phosphorylation level and the number of existing casein isoforms important for milk coagulation.

Another post-translational modification in κ-CN is the glycosylation of Thr residues. This occurs for Thr near Arg/Lys, Thr or Pro, and is likely to be inhibited by Ile [45]. Based on this information we propose glycosylation of four Thr residues at 109, 127, 149 and 153 (Fig 4). This finding agrees with the analysis of Kappeler et al. [15] on dromedary camel, however no further information are available in llamas so far, also in regard of glycosylated and non-glycosylated forms of GMP, therefore future investigations in this field are desirable.

### Inter-specific genetic variability

Inter-specific genetic variability was investigated by the comparison of the lama casein cDNA genes with the published sequences of the camel cDNA [15].

A total of 96 polymorphic sites were found (Table 2). Fifty one out of 96 SNPs (53.12%) fell in coding regions and they are responsible of 22 amino acid changes in total. In addition, 36 SNPs were detected in 3’ UTR (37.50%) and 9 in 5’ UTR (9.37%).

**Table 2:** Genetic variability detected by the comparison among the casein genes transcripts in llama and the dromedary camel cDNAs reported by Kappeler et al. [15].

*CSN1S1* (αs1-casein)				*CSN2* (β-casein)				*CSN1S2* (αs2-casein)				*CSN3* (κ-casein)			
Exon	Position	Llama	Dromedary	Exon	Position	Llama	Dromedary	Exon	Position	Llama	Dromedary	Exon	Position	Llama	Dromedary
	cDNA^aa^	LK999986	AJ012628		cDNA^aa^	LK999992	AJ012630		cDNA^aa^	LK999989	AJ012629		cDNA^aa^	LK999995	Y10082
1	51	G	a	1	45–46	ga	—	2	66^–14^	*AA* *G* ^Lys^	*AA* *A* ^Lys^	1	21	a	g
2	58	c	t	1	50–51	aa	tt	2	98^–3^	GCT^**Ala**^	GTT^**Val**^	1	50	g	a
2	98^–5^	*GC* *C* ^Ala^	*GG* *T* ^Ala^	2	60	t	g	5	168^21^	TTA ^**Leu**^	TTT ^**Phe**^	3	135^–14^	*GT* *C* ^Val^	*GT* *T* ^Val^
4	168^20^	CAG^**Gln**^	GAG^**Glu**^	2	90^–7^	*C* *T* *G* ^**Leu**^	*C* *G* *G* ^**Arg**^	6	222^39^	ACG ^Thr^	ACA ^Thr^	3	159^–6^	*CT* *G* ^Leu^	*CT* *A* ^Leu^
10	307^66^	ACG^**Thr**^	AAG^**Lys**^	3	136^9^	GCG ^Ala^	GCC ^Ala^	6	228^41^	TGT ^Cys^	TGC ^Cys^	5	204^10^	TGC ^Cys^	TGT ^Cys^
10	310^67^	GTA^**Val**^	GAA^**Glu**^	4	146^13^	GTG^**Val**^	TTG^**Leu**^	8	266^54^	GTT^**Val**^	GCT^**Ala**^	5	206^11^	TGT^**Cys**^	TTT^**Phe**^
10	323^71^	AGC ^Ser^	AGT ^Ser^	7	310^67^	ATT ^Ile^	ATC ^Ile^	9	357^84^	GTT ^Val^	GTG ^Val^	5	216^14^	GTA ^Val^	GTG ^Val^
12	396–397^96^	ATT^**Ile**^	TAT^**Tyr**^	7	343^78^	CCT ^Pro^	CCG ^Pro^	9	363^86^	AAT ^Asn^	AAC ^Asn^	5	264^30^	TTT ^Phe^	TTC ^Phe^
15	482^124^	AAA ^Lys^	AAG ^Lys^	7	414^102^	ATC^**Ile**^	ACC^**Thr**^	9	386^94^	ATG^**Met**^	AGG^**Arg**^	5	348^58^	TAT ^Tyr^	TAC ^Tyr^
15	483^125^	TTG^Leu^	CTG^Leu^	7	416^103^	GTC^**Val**^	ATC^**Ile**^	9	389^95^	GTC^**Val**^	GCC^**Ala**^	5	360^62^	GCA ^Ala^	GCC ^Ala^
19	604^165^	CAA^**Gln**^	CCA^**Pro**^	7	458^117^	CTA^**Leu**^	GTA^**Val**^	13	501^132^	GAG ^Glu^	GAA ^Glu^	5	450^92^	CGT ^Arg^	CGC ^Arg^
19	690^194^	CAC^**His**^	TAC^**Tyr**^	7	545^146^	CAC^**His**^	TAC^**Tyr**^	17	719	t	g	5	506^111^	ATC^**Ile**^	AAC^**Asn**^
21	814	a	-	7	680^191^	CTT^**Leu**^	GTT^**Val**^	17	762	a	g	5	510^112^	CCC ^Pro^	CCT ^Pro^
21	821	a	g	7	701^198^	ATA^**Ile**^	GTA^**Val**^	17	766–767	ca	tg	5	537^121^	GAA ^Glu^	GAG ^Glu^
21	838	t	c	7	745^212^	CTC ^Leu^	CTT ^Leu^	17	771	g	a	5	546^124^	GTC ^Val^	GTT ^Val^
21	842	t	c	9	826	c	a	17	777	g	a	5	558^128^	GCT ^Ala^	GCC ^Ala^
21	883	a	g		857	t	c	17	912/913	-	t	5	566^131^	GTA^**Val**^	GCA^**Ala**^
21	988	a	c		869	g	a	17	965	g	a	5	618^148^	AGT ^Ser^	AGC ^Ser^
21	1172	c	t		871/872	-	t					5	651^159^	TCA ^Ser^	TCG ^Ser^
					882	g	a					5	678	t	g
					885	g	a					6	730	t	c
					892	t	c								
					900	c	t								
					972	a	g								
					987	t	g								
					990	g	a								
					994	c	t								
					1003	t	c								
					1021	a	g								
					1023	g	t								
					1030/1031	-	a								
					1045	g	t								
					1065	g	a								
					1089	t	c								

Polymorphic nucleotides in the triplets are underlined, when they fall within the peptide leader they are also reported in Italics. Amino acid changes are showed in bold. Insertions/deletions are reported as dashes. Numbering is relative to the llamas cDNAs.

The most polymorphic cDNA was the *CSN2*, counting 36 polymorphic sites and a total of 8 amino acid changes (Table 2), which resulted in a slightly higher predicted isoelectric point (*pI* = 5.30) for the mature llama β-casein in respect of the dromedary protein (*pI* = 5.15).

On the contrary, the most conserved cDNA was the *CSN1S2* with 19 polymorphic sites responsible for 5 amino acid changes. Twenty SNPs and 6 amino acid replacements were counted for the *CSN1S1*, whereas 21 SNPs responsible for 3 amino acids changes were detected for the *CSN3* cDNA (Table 2). Lower *pI* were predicted for the llama αs2- (5.55) and κ-casein (7.95) in comparison with dromedary proteins (5.75 and 8.20 respectively), whereas the αs1-casein showed a very similar value (4.85 *vs* 4.80), most probably as result of a compensative effect of the charges between the 6 amino acids changes.

Many genetic variants of caseins have been identified so far at the DNA or protein level in domestic animals and analysed for different aspects including allergenicity to humans [46]. South American camelids (llamas and alpacas) were not investigated so far, whereas recently two alleles (*CSN2* A and *CSN2* B) were identified in bactrian camels β-casein [9], a new allele (*CSN1S1* C) was characterized at protein and DNA level of dromedary camel by Shuiep et al. [10] and several polymorphisms were also found in the *CSN3* gene [8], thus increasing the interest for the most represented milk proteins in Camelidae.

### Analysis of the regulatory flanking regions

The analysis of the genes flanking regions (FRs) provides an important contribution for the evaluation of the transcription factors involved in the regulation of the gene expression. For this reason, we decided to extend the sequencing of the llama casein genes to the 5’- and 3’-FRs and to analyze the putative transcription factors.

The sequences of the 5’-FRs for the *CSN1S1*, *CSN2*, *CSN1S2* and *CSN3* were submitted to EMBL with the following IDs: LK999985, LK999991, LK999988, LK999994; whereas the sequences corresponding to the 3’-FRs of the same genes were submitted with the following IDs: LK999987, LK999993, LK999990, LK999996.

A total of 505 high-scoring (85%-100%) putative binding sites were found by TFSERCH tool. The most representative consensus sequences related to protein and milk production identified in the proximal promoter region were those belonging to the octamer binding family (Oct), GATA-binding proteins, C/EBPs (CCAAT enhancer binding proteins), ubiquitous activators like AP-1, AP-2, SP1, etc. In Table 3 we report the consensus sequences common to the four casein and showing the higher binding scores.

**Table 3:** Most representative consensus motifs for transcription factors detected in the 5’-flanking regions of llamas by TFSEARCH software and present in all caseins with higher binding score (BS).

Transcription factor	Consensus motif	*CSN1S1*			*CSN2*			*CSN1S2*			*CSN3*		
		Position	S	BS	Position	S	BS	Position	S	BS	Position	S	BS
AML1/Runx	TGTGGT	-259/-254	-	0.873	-57/-52	-	1.000	-300/-295	+	0.910	-61/-56	-	0.850
AP-1	RSTGACTNMNW	-186/-176	-	0.850				-98/-88	+	0.851	-104/-94	-	0.890
C/EBP	NNTKTGGWNANNN	-304/-292	-	0.940	-271/-259	+	0.911	-51/-39	-	0.927	-58/-45	-	0.875
GATA	NNNGATRNNN	-106/-97	+	0.870	-183/-174	+	0.887	-350/-341	-	0.860	-124/-115	-	0.931
HNF3	NNNTRTTTRYTY	-83/-72	+	0.880	-77/-66	+	0.928	-338/-327	+	0.928	-20/-9	+	0.932
MyoD	SRACAGGTGKYG				-307/-296	+	0.874	-265/-254	+	0.925	-62/-51	-	0.858
Oct-1	NNNRTAATNANNN	-267/-255	-	0.929	-131/-120	+	0.917	-186/-174	+	0.947	-84/-72	+	0.852
Pbx-1	ANCAATCAW	-45/-37	+	0.942	-109/-101	+	0.903	-221/-213	+	0.899	-33/-25	-	0.912
SRY	AAACWAM	-253/-248	+	0.941	-192/-186	-	0.960	-169/-163	+	0.947	-14/-8	-	0.939
MGF/STAT5	TTCCCRKAA	-270/-262	+	0.931	-94/-86	-	0.870	-292/-284	-	0.956			
TATA-box	WTATAAAW	-31/-25	+	0.980	-28/-19	+	0.910	-23/-16	+	0.865	-18/-11	-	0.927

DNA strands (S) in direction 5’>3’ are indicated by +.

The opposite strands are indicated by-.

Positive and negative regulators of casein gene expression were found. In particular, 33 putative C/EBP elements and 11 Mammary Gland Factor/STAT5 (MGF/STAT5) were found. These elements are known to be activators of transcription through sinergyc interactions [47]. Sixty-three consensus sequences for Octamer bind protein (Oct-1) were found, all potentially involved in the activation of the llama casein genes transcription. Oct-1 is not known to be a strong transcriptional activator by itself, but in conjunction with other co-activators. For instance, Zhao et al. [48] showed that Oct-1 and STAT5 are both involved in the hormonal induction of casein gene expression. Oct-1 can also interact with acute myeloid leukemia factors (AML, also known as Runx) by relieving the auto-inhibitory function of the Runx DNA binding and forming a complex which activate the expression of casein genes [49].

The presence of hepatocyte nuclear factors-3 (HNF3) instead confirms the potential activation of the gene expression by the interaction with adjacents C/EBP and glucocorticoid elements (GR) [50, 51]. Similar interactions are supposed also for the MyoD in the coindution of retinoblastoma tumor soppressor and β-casein for milk genes expression [52] and for Pbx1 in the synergic cross-talk with glucocorticoid receptors [53]. Several other transcription factors were identified including SRY, CdxA, CRE-BP, MZF1, SP1, NF- YY1, etc. as was already described in dromedary camel promoters [8, 9, 54].

Furthermore, it is interesting to underline the presence of one sterol regulatory element binding protein (SREBP) at position (-61/-51) of the κ-casein promoter. In cattle, this element is considered a key component in the regulation of milk fat synthesis [55] and, to our knowledge, it was never reported to control the expression of casein genes. However, recently Reed et al. [56] revealed novel functional role of SREBP in the regulation of distinct classes of genes, including the β- (-0,015), κ- (-0,129) and αs1-casein (-0,156) differently down-regulated by SREBP.

The 3’-flanking regions instead were characterized by a lower number of consensus sequences. They belong to the same groups of transcription factors. In particular C/EBP-α elements were found in *CSN2*, *CSN1S2* and *CSN3*, GR and SP1 motifs at the αs1- and αs2-casein genes, whereas Oct-1 were detected only at the *CSN3*.

## Conclusion

Casein genes have been deeply investigated in ruminants, whereas little information is available in camelids and, so far, llama was never investigated. In this study we propose for the first time a full characterization of the complete casein cluster through the transcripts analysis of the four llama casein genes. The sequence of the correctly assembled transcripts allowed us to establish the exonic structure of each gene.

Exons skipping and duplication events were evidenced. We were able to identify two variants A (215 amino acids) and B (207 amino acids) in the αs1-casein gene as result of the alternative out-splicing of the exon 18, which opens the possibility for further genetic studies. No splicing were evidenced in the β- and αs2-casein, although in the latter one extra exon was found through the comparison with the dromedary camel sequence. A cryptic exon of 43 bp was found in the κ-casein. This finding is particularly interesting because it leads to have two possible ATG (Methionine) as translation start codon.

The characterization at cDNA level of llama casein is fundamental for the detection of genetic variability, for studies involving the identification of protein variants as well as post translational modifications. In this regard, the differences predicted in the *pI* might be responsible for different migration patterns for the llama casein fractions in iso-electro-focusing (IEF) experiments. Therefore, the information herein reported might be helpful to establish new IEF standards in llama and useful for the identification of protein variants.

This work adds fundamental knowledge on the molecular basis of milk protein genes in llamas and can be useful for the understanding of the evolution of casein genes especially in Camelidae. Future studies will allow to figure out the functional characteristics (nutritional and dietetic) of this milk to better meet the food requirements of rural population, but also for the newborn offspring.

## References

[1] Trinks A, Burger PA, Beneke N, Burger J. Simulation of populations ancestry of the two-humped camel (*Camelus bactrianus*) In: Knoll EM, Burger PA, editors. Camels in Asia and North Africa. Interdisciplinary perspectives on their significance in past and present. Vienna: Austrian Academy of Sciences Press; 2012 p. 79–86.

[2] Allain D, Renieri C. Genetics of fibre production and fleece characteristics in small ruminants, Angora rabbit and South American camelids. Animal. 2010;4:1472–81. 10.1017/S1751731110000029 22444694

[3] Rijnkels M. Multispecies comparison of the casein gene loci and evolution of casein gene family. J Mammary Gland Biol Neoplasia. 2002;7:327–345. 12751895 10.1023/a:1022808918013

[4] Caroli A, Chiatti F, Chessa S, Rignanese D, Bolla P, Pagnacco G. Focusing on the goat casein complex. J Dairy Sci. 2006;89:3178–87. 16840635 10.3168/jds.S0022-0302(06)72592-9

[5] Cosenza G, Pauciullo A, Gallo D, Colimoro L, D’avino A, Mancusi A, Ramunno L. Genotyping at the *CSN1S1* locus by PCR-RFLP and AS-PCR in a Neapolitan goat population. Small Rumin Res. 2008;74:84–90.

[6] Caroli AM, Chessa S, Erhardt GJ. Milk protein polymorphisms in cattle: effect on animal breeding and human nutrition. J. Dairy Sci. 2009;92:5335–52. 10.3168/jds.2009-2461 19841193

[7] Ramunno L, Cosenza G, Rando A, Pauciullo A, Illario R, Gallo D, et al Comparative analysis of gene sequence of goat *CSN1S1* F and N alleles and characterization of *CSN1S1* transcript variants in mammary gland. Gene. 2005;345:289–299. 15716101 10.1016/j.gene.2004.12.003

[8] Pauciullo A, Shuiep ES, Cosenza G, Ramunno L, Erhardt G. Molecular characterization and genetic variability at κ-casein gene (*CSN3*) in camels. Gene. 2013;513:22–30. 10.1016/j.gene.2012.10.083 23154061

[9] Pauciullo A, Giambra IJ, Iannuzzi L, Erhardt G. The β-casein in camels: molecular characterization of the *CSN2* gene, promoter analysis and genetic variability. Gene. 2014;547:159–68. 10.1016/j.gene.2014.06.055 24973699

[10] Shuiep ES, Giambra IJ, Yas Mohamed El Zubeir IE, Erhardt G. Biochemical and molecular characterization of polymorphisms of αs1-casein in Sudanese camel (*Camelus dromedarius*) milk. Int Dairy J. 2012;28:88–93.

[11] Martin P, Leroux C. Exon-skipping is responsible for the 9 amino acid residue deletion occurring near the N-terminal of human beta-casein. Biochem Biophys Res Commun. 1992;183:750–7. 1550581 10.1016/0006-291x(92)90547-x

[12] Riek A, Gerken M Changes in Llama (*Lama glama*) milk composition during lactation. J Dairy Sci. 2006;89:3484–93. 16899683 10.3168/jds.S0022-0302(06)72387-6

[13] Riek A, Klinkert A, Gerken M, Hummel J, Moors E, Südekum KH. Short communication: milk output in llamas (*Lama glama*) in relation to energy intake and water turnover measured by an isotope dilution technique. J Dairy Sci. 2013;96:1815–9. 10.3168/jds.2012-6323 23332845

[14] Saadaoui B, Bianchi L, Henry C, Miranda G, Martin P, Cebo C. Combining proteomic tools to characterize the protein fraction of llama (*Lama glama*) milk. Electrophoresis. 2014;35:1406–18. 10.1002/elps.201300383 24519815

[15] Kappeler S, Farah Z, Puhan Z. Sequence analysis of Camelus dromedarius milk caseins. J Dairy Res. 1998;65:209–22. 9627840 10.1017/s0022029997002847

[16] Tagliabracci VS, Engel JL, Wen J, Wiley SE, Worby CA, Kinch LN, et al Secreted kinase phosphorylates extracellular proteins that regulate biomineralization. Science. 2012;336:1150–1153. 10.1126/science.1217817 22582013 PMC3754843

[17] Ferranti P, Malorni A, Nitti G, Laezza P, Pizzano R, Chianese L, et al Primary structure of ovine αs1-caseins: localization of phosphorylation sites and characterization of genetic variants A, C and D. J Dairy Res. 1995;62:281–96. 7601973 10.1017/s0022029900030983

[18] Leroux C, Mazure N, Martin P. Mutations away from splice site recognition sequences might cis-modulate alternative splicing of goat as1-casein transcripts. Structural organization of the relevant gene. J Biol Chem. 1992;267:6147–57. 1372900

[19] Mohr U, Koczan D, Linder D, Hobom G, Erhardt G. A single point mutation results in A allele-specific exon skipping in the bovine alpha s1-casein mRNA. Gene. 1994;143:187–92. 8206372 10.1016/0378-1119(94)90095-7

[20] Alexander LJ, Das Gupta NA, Beattie CW. The sequence of porcine αs1-casein cDNA. Anim Genet. 1992;23:365–67. 1503276

[21] Johnsen LB, Rasmussen LK, Petersen TE, Berglung L. Characterization of three types of human αs1-casein mRNA transcripts. Biochem J. 1995;309:237–42. 7619062 10.1042/bj3090237 PMC1135825

[22] Martin P, Szymanowska M, Zwierzchowski L, Leroux C. The impact of genetic polymorphisms on the protein composition of ruminant milks. Reprod Nutr Dev. 2002;42:433–459. 12537255 10.1051/rnd:2002036

[23] Smith CW, Chu TT, Nadal-Ginard B. Scanning and competition between AGs are involved in 3’ splice site selection in mammalian introns. Mol Cell Biol. 1993;13:4939–52. 8336728 10.1128/mcb.13.8.4939-4952.1993 PMC360135

[24] Jones WK, Yu-Lee LY, Clift SM, Brown TL, Rosen IM. The rat casein multigene family. Fine structure and evolution of the β-casein gene. J Biol Chem. 1985;260:7042 3997858

[25] Bonsing J, Mackinlay AG. Recent studies on nucleotide sequences encoding the caseins. J Dairy Res. 1987;54:447–61. 3308989 10.1017/s0022029900025632

[26] Ramunno L, Cosenza G, Rando A, Illario R, Gallo D, Di Berardino D, et al The goat alpha s1-casein gene: gene structure and promoter analysis. Gene. 2004;334:105–11. 15256260 10.1016/j.gene.2004.03.006

[27] Mercier JC, Vilotte JL. Structure and function of milk protein genes. J Dairy Sci. 1993;76:3079–98. 8227632 10.3168/jds.S0022-0302(93)77647-X

[28] Cosenza G, Pauciullo A, Annunziata AL, Rando A, Chianese L, Marletta D, et al Identification and characterization of the donkey *CSN1S2* I and II cDNAs. Ital J Anim Sci. 2010;9:e40.

[29] Boisnard M, Hue D, Bouniol C, Mercier JC, Gaye P. Multiple mRNA species code for two non-allelic forms of ovine as2-casein. Eur J Biochem. 1991;201:633–41. 1935959 10.1111/j.1432-1033.1991.tb16324.x

[30] Bouniol C, Printz C, Mercier JC. Bovine alpha s2-casein D is generated by exon VIII skipping. Gene. 1993;128:289–93. 8514196 10.1016/0378-1119(93)90577-p

[31] Ramunno L, Longobardi E, Pappalardo M, Rando A, Di Gregorio P, Cosenza G, et al An allele associated with a non-detectable amount of αs2 casein in goat milk. Anim Genet. 2001;32:19–26. 11419340 10.1046/j.1365-2052.2001.00710.x

[32] Cosenza G, Pauciullo A, Feligini M, Coletta A, Colimoro L, Di Berardino D, et al A point mutation in the splice donor site of intron 7 in the as2-casein encoding gene of the Mediterranean River buffalo results in an allele-specific exon skipping. Anim Genet. 2009;40:791 10.1111/j.1365-2052.2009.01897.x 19422363

[33] Fujita H, Sugimoto K, Inatomi S, Maeda T, Osanai M, Uchiyama Y, et al Tight junction proteins claudin-2 and -12 are critical for vitamin D-dependent Ca^2+^ absorption between enterocytes. Mol Biol Cell. 2008;19:1912–21. 10.1091/mbc.E07-09-0973 18287530 PMC2366872

[34] Chin D, Means AR. Calmodulin: a prototypical calcium sensor. Trends Cell Biol. 2000;10:322–328. 10884684 10.1016/s0962-8924(00)01800-6

[35] Ottenheijm CA, Fong C, Vangheluwe P, Wuytack F, Babu GJ, Periasamy M, et al Sarcoplasmic reticulum calcium uptake and speed of relaxation are depressed in nebulin-free skeletal muscle. FASEB J. 2008;22:2912–9. 10.1096/fj.07-104372 18434434 PMC2493448

[36] Halper J, Kjaer M. Basic components of connective tissues and extracellular matrix: elastin, fibrillin, fibulins, fibrinogen, fibronectin, laminin, tenascins and thrombospondins. Adv Exp Med Biol. 2014;802:31–47. 10.1007/978-94-007-7893-1_3 24443019

[37] Jollès P, Loucheux-Lefebvre MH, Henschen A. Structural relatedness of κ-casein and fibrinogen γ-chain. J Mol Evol. 1978;11:271–277. 722804 10.1007/BF01733837

[38] Clark F, Thanaraj TA. Categorization and characterization of transcript-confirmed constitutively and alternatively spliced introns and exons from human. Hum Mol Genet. 2002;11:451–64. 11854178 10.1093/hmg/11.4.451

[39] Zhang MQ. Computational prediction of eukaryotic protein-coding genes. Nat Rev. 2002;3:698–710. 12209144 10.1038/nrg890

[40] Menon RS, Chang YF, Jeffers KF, Ham RG. Exon skipping in human β-casein. Genomics. 1992;12:13–17. 1370811 10.1016/0888-7543(92)90400-m

[41] Menon RS, Chang YF, Ham RG. Reversal of exon skipping in human β-casein following restoration of an uninterrupted polypyrirnidine tract. Mol Biol Cell. 1992;3:323 1627832

[42] Holland JW. Post-translational modifications of caseins. In: Abby Thompson editor. Milk protein from expression to food. Amsterdam, Netherlands. 2009. p. 107–132.

[43] Mahé MF, Grosclaude F. Polymorphisme de la caséine α(s2) des Bovinés: caractérisation du variant C du Yak (*Bos grunniens*). Ann Genet Sel Anim. 1982;14:401–16. 10.1186/1297-9686-14-4-401 22896248 PMC2734573

[44] Li S, Wang J, Wie H, Yang Y, Bu D, Zhang L, et al Identification of bovine casein phosphorylation using titanium dioxide enrichment in combination with nano electrospray ionization tandem mass spectrometry. J Integr Agr. 2012;11:439–445. 10.1142/S0219635212500288 23351051

[45] Pisano A, Packer NH, Redmond JW, Williams KL, Gooley AA. Characterisation of O-linked glycosylation motifs in the glycopeptide domain of bovine κ-casein. Glycobiology 1994;4:837–844. 7734846 10.1093/glycob/4.6.837

[46] Lisson M, Novak N, Erhardt G. Immunoglobulin E epitope mapping by microarray immunoassay reveals differences in immune response to genetic variants of caseins from different ruminant species. J Dairy Sci. 2014;97:1939–54. 10.3168/jds.2013-7355 24485684

[47] Wyszomierski SL, Rosen JM. Cooperative effects of STAT5 (signal transducer and activator of transcription 5) and C/EBP beta (CCAAT/ enhancer-binding protein-beta) on beta-casein gene transcription are mediated by the glucocorticoid receptor. Mol Endocrinol. 2001;15:228–240. 11158330 10.1210/mend.15.2.0597

[48] Zhao FQ, Adachi K, Oka K. Involvement of Oct-1 in transcriptional regulation of β-casein gene expression in mouse mammary gland. Biochim Biophys Acta. 2002;1577:27–37. 12151092 10.1016/s0167-4781(02)00402-5

[49] Inman CK, Li N, Shore P. Oct-1 Counteracts Autoinhibition of Runx2 DNA Binding To Form a Novel Runx2/Oct-1 Complex on the Promoter of the Mammary Gland-Specific Gene β-casein. Mol Cell Biol. 2005;25:3182–93. 15798204 10.1128/MCB.25.8.3182-3193.2005 PMC1069618

[50] Schild TA, Geldermann H. Variants within the 5'-flanking regions of bovine milk-protein-encoding genes. III. Genes encoding the Ca-sensitive caseins αs1, αs2 and β. Theor Appl Genet. 1996;93:887–893. 10.1007/BF00224090 24162422

[51] Christoffels VM, Grange T, Kaestner KH, Cole TJ, Darlington GJ, Croniger CM, et al Glucocorticoid receptor, C/EBP, HNF3, and protein kinase A coordinately activate the glucocorticoid response unit of the carbamoylphosphate synthetase I gene. Mol Cell Biol. 1998;18:6305–15. 9774647 10.1128/mcb.18.11.6305 PMC109217

[52] Jiang Z, Zacksenhaus E. Activation of retinoblastoma protein in mammary gland leads to ductal growth suppression, precocious differentiation, and adenocarcinoma. J Cell Biol. 2002;156:185–98. 11777937 10.1083/jcb.200106084 PMC2173568

[53] Subramaniam N, Campión J, Rafter I, Okret S. Cross-talk between glucocorticoid and retinoic acid signals involving glucocorticoid receptor interaction with the homoeodomain protein Pbx1. Biochem J. 2003;370:1087–95. 12487626 10.1042/BJ20020471 PMC1223238

[54] Kappeler SR, Farah Z, Puhan Z. 5’-Flanking regions of camel milk genes are highly similar to homologue regions of other species and can be divided into two distinct groups. J Dairy Sci. 2003;86:498–508. 12647956 10.3168/jds.S0022-0302(03)73628-5

[55] Harvatine KJ, Bauman DE. SREBP1 and thyroid hormone responsive spot 14 (S14) are involved in the regulation of bovine mammary lipid synthesis during diet-induced milk fat depression and treatment with CLA. J Nutr. 2006;136:2468–74. 16988111 10.1093/jn/136.10.2468

[56] Reed BD, Charos AE, Szekely AM, Weissman SM, Snyder M. Genome-wide occupancy of SREBP1 and its partners NFY and SP1 reveals novel functional roles and combinatorial regulation of distinct classes of genes. PLoS Genet. 2008;4:e1000133 10.1371/journal.pgen.1000133 18654640 PMC2478640

